# Intergroup statement: opportunistic salpingectomy—molecular pathology, clinical outcomes and implications for practice (German Ovarian Cancer Commission, the North-Eastern German Society of Gynecologic Oncology (NOGGO), AGO Austria and AGO Swiss)

**DOI:** 10.1007/s00404-025-07974-z

**Published:** 2025-04-02

**Authors:** Martin Pölcher, Pauline Wimberger, Ivo Meinhold-Heerlein, Ingo Runnebaum, Susanne Schüler-Toprak, Sven Mahner, Christoph Grimm, Viola Heinzelmann-Schwarz, Annette Hasenburg, Jalid Sehouli

**Affiliations:** 1 Department of Gynecologic Oncology and Minimally Invasive Surgery, Rotkreuzklinikum München Frauenklinik, Taxisstraße 3, 80637 Munich, Germany; 2 Department of Gynecology and Obstetrics, Technische Universität Dresden, Dresden, Germany; 3 Department of Gynecology and Obstetrics, Justus Liebig University Giessen, Giessen, Germany; 4 Department of Gynecology and Reproductive Medicine, Jena University Hospital, Friedrich Schiller-University Jena, Jena, Germany; 5 Department of Gynecology and Obstetrics, University Medical Center Regensburg, Regensburg, Germany; 6 Department of Obstetrics and Gynaecology, University Hospital, Ludwig-Maximilians-University Munich, 81377 Munich, Germany; 7 Department of Obstetrics and Gynecology, Medical University of Vienna and Comprehensive Cancer Center, Vienna, Austria; 8 Gynecological Cancer Center, Women’s Hospital, University Hospital Basel, Basel, Switzerland; 9 Obstetrics and Gynecology, Mainz University, Mainz, Germany; 10 Department of Gynecology with Center for Oncological Surgery, Charité-University Medicine Berlin, Campus Virchow-Klinikum, Berlin, Germany; 11 Ovarian Cancer Research, Department of Biomedicine, University of Basel, Basel, Switzerland

**Keywords:** Prevention, Ovarian cancer, Opportunistic salpingectomy, STIC

## Abstract

Opportunistic salpingectomy is defined as the removal of both fallopian tubes as part of a surgical procedure planned for other reasons. The goal is primary prevention of ovarian cancer. The procedure is offered to patients who are not known to be at increased risk of developing ovarian cancer. This is in contrast to high-risk patients with a germline mutation, particularly BRCA1/2, for whom risk-reducing salpingo-oophorectomy is generally recommended. Premalignant cells and early occult cancers have been detected in RRSO specimens in the fimbrial funnel region, but not on the ovarian surface. The presence of mitoses, nuclear atypia, and staining in response to p53 mutation in these serous intraepithelial carcinomas (STIC) indicates the initial genetic changes in the fallopian tube mucosa that subsequently lead to the development of advanced peritoneal carcinomas. The identification of STICs has challenged the traditional view of the pathogenesis of the largest subset of epithelial ovarian cancers, namely the high-grade serous cancers of the ovary, fallopian tubes, and peritoneum. In a position statement published in 2015, the German Arbeitsgemeinschaft Gynäkologische Onkologie (AGO) Kommission Ovar recommended that patients be informed of the latest findings on the development and potential benefits of bilateral salpingectomy at the time of hysterectomy. This may reduce the risk of developing ovarian cancer later in life. However, the scientific evidence has not been deemed sufficient to justify a general recommendation. In the same year, the Austrian AGO published a statement recommending the broad use of opportunistic salpingectomy without reservation. This review examines the current status of molecular pathology studies, recent evidence on the clinical implications of STIC, new data on the use of opportunistic salpingectomy, and published patient outcomes since then. The question of whether the potential benefit of opportunistic salpingectomy, outweighs the potential harms associated with surgical morbidity, which have not been conclusively excluded, should be revisited in light of these recent data.

**Supplementary Information:**

The online version contains supplementary material available at 10.1007/s00404-025-07974-z.

## Opportunistic salpingectomy—a strategy for cancer prevention?

Worldwide, an estimated 324,000 new cases of ovarian cancer are diagnosed each year, resulting in approximately 207,000 deaths from the disease. This means that ovarian cancer has the highest mortality rate of all gynecological cancers [1]. Malignant ovarian tumors can be classified according to their origin as germ cell tumors, germinal-strand stromal tumors, or surface epithelial ovarian carcinomas. The latter are further subdivided into five distinct morphologic subtypes: Brenner, mucinous, clear cell, endometrioid, and serous. Serous carcinomas can be further subdivided into low-grade and high-grade serous tubal and ovarian carcinoma (HGSTOC).

The largest group of malignant ovarian tumors, accounting for 70–80% of all carcinomas, are HGSTOC, which also correlate with the worst prognosis. Individuals with a BRCA1/2 mutation, who are at high risk of developing ovarian cancer, typically present with HGSTOC. Despite the premature onset of menopause and the associated adverse effects on e.g. sexual function, bone and cardiovascular health, primary prevention with risk-reducing salpingo-oophorectomy (RRSO) remains the gold standard of care for carriers of pathogenic variants in BRCA1 or BRCA2 or other mutations such as RAD51C, PALB2 and CHEK2 [2].

However, the majority of HGSTOCs occur in an average-risk population, with approximately 75% of these carcinomas developing sporadically and showing no discernible association with genetic predisposition [3].

A significant clinical challenge remains the delayed diagnosis at advanced stages. Despite numerous attempts at early detection through various screening methods, no effective approach has been established [4]. A recent study found that ultrasound and serial tumor marker testing resulted in a staging shift to earlier tumor stages, but did not reduce mortality [5]. Currently, no effective screening methods exist for the prevention or early clinical detection of HGSTOC.

The identification of STICs in the fallopian tubes and the reduction in the risk of ovarian cancer in patients who have undergone tubal ligation or salpingectomy, as evidenced by epidemiologic data, suggest a potential primary prevention strategy in which bilateral salpingectomy is performed instead of tubal ligation and bilateral opportunistic salpingectomy is consistently performed during abdominal surgery, once the family planning process has been completed. Following an initial comprehensive campaign by the Society of Gynecologic Oncologists of Canada (OVCARE) in 2011 [6], guidelines, clinical practice statements, and recommendations for opportunistic salpingectomy were adopted by numerous national professional associations around the world, including the German AGO-Commission OVAR and the Austrian AGO in 2015. [7, 8].

## Frequency of precursor lesions

In recent years, pathologic and molecular genetic studies have focused on the fimbrial funnel, where changes in secretory cells have been described that represent a stepwise progression from tubal epithelial cells to carcinoma [9]. This progression can be observed in cells with a so-called p53 signature, which then develop into serous intraepithelial lesions (STIL) and finally into serous intraepithelial carcinoma (STIC) [10].

The increasing number of somatic mutations when comparing p53 signatures with STIC and HGSTOC, coupled with the increasing rate of loss of heterozygosity (LOH), is consistent with the concept of stepwise tumorigenesis [11]. Transcriptional analyses indicate that HGSTOC have a higher degree of similarity to fallopian tube epithelium than to ovarian epithelium or peritoneal mesothelial cells [12, 13]. A significant proportion of STICS have identical TP53 mutations as their coexisting HGSTOC [14, 15]. Furthermore, the methylation profile of a STIC is more closely matched to that of the associated HGSTOC than to the surrounding fimbrial cells: the genomic trajectory of ovarian high-grade serous carcinoma can be observed in STIC lesions [16].

In specimens from risk-reducing salpingo-oophorectomy (RRSO) in BRCA1/2 germline mutation carriers, a p53 signature is observed in 27% of cases and STIC in up to 10% after special processing of the tube (Sectioning and Extensively Examining the Fimbriated End-SEE-FIM protocol [17]) with immunohistochemical staining for p53 and Ki67. In contrast, in the average-risk general population, i.e., women without pathogenic germline mutations in BRCA1/2 or other homologous recombination genes, tubal lesions are extremely rare (less than 0.1%). Examination of an existing HGSTOC will reveal the presence of STICs in approximately half of the cases when appropriately processed using the SEE-FIM protocol [18]. This also shows that the number of identified precursors depends to a large extent on the effort put into processing the specimen [19].

Though STICs are associated with a significantly increased risk of HGSTOC, not all will progress to malignant neoplasms. For clinical follow-up after diagnosis of STICs, further differentiation would be beneficial to identify which patients will develop peritoneal carcinomatosis and which will not. Recently, different morphologic patterns of precursor lesions have been identified, including those with a flat surface and those with detached cellular components (budding, loosely adherent, or detached; BLAD). The latter may be associated with a less favorable prognosis due to additional features, including CCNE1 amplification and specific aneuploidy patterns. Of particular concern is their higher association with concurrent invasive HGSC. These growth forms may provide a basis for further correlation studies to stratify tubal precursor lesions [20].

## Clinical implications of a diagnosis of STIC

In the subsequent analysis of patients with a mutation in BRCA 1/2 and indications of isolated STIC, a notable proportion were diagnosed with serous carcinomatosis within a 10-year period. (21.6% or 33.9%, depending on the study [21, 22]). In addition, the presence of STIC without evidence of invasive carcinoma may be associated with synchronous invasive serous carcinoma in the peritoneum or lymph nodes. The potential benefit of surgical staging in patients with identified STICs remains to be demonstrated. In 2020, all German gynecologic centers were invited to participate in a 21-question questionnaire designed to explore clinical experience with STICs and the associated diagnostic, surgical, and histopathologic approaches. The results revealed significant inconsistencies in how these incidental findings get managed [23]. In view of these findings, a German STIC registry was initiated in October 2024, with the objective of gathering clinical and histopathologic data on these patients. The registry will include all patients diagnosed with isolated STIL or STIC, both retrospectively and prospectively, with annual follow-ups conducted to provide a basis for future clinical guidelines.

## The extent of ovarian cancer risk reduction by tubal ligation or by bilateral salpingectomy

A Swedish population-based retrospective cohort study was recently published, shortly after the release of the 2015 AGO Statement. The study evaluated data from the National Health Register to identify 2,514,665 patients who underwent hysterectomy, hysterectomy with bilateral adnexectomy, salpingectomy, or tubal sterilization for benign conditions between 1973 and 1996. The analysis revealed a total of 30,749 cases of ovarian cancer diagnosed during the observation period, which concluded in 2009, with a median follow-up period of 23.1 years. A subsequent comparison of the risk of developing ovarian cancer between patients who had undergone hysterectomy and those who had not revealed a statistically significant reduction in the former group (hazard ratio (HR) = 0.79, 95% confidence interval (CI) = 0.70 to 0.88). Additionally, a reduction in the risk of ovarian cancer was observed in patients who had undergone sterilization (HR = 0.72, 95% CI 0.64 to 0.81) and in patients who had undergone hysterectomy and bilateral adnexectomy (HR = 0.06, 95% CI 0.03 to 0.12). In comparison to unilateral surgery, bilateral salpingectomy demonstrated a 50% reduction in the risk of developing ovarian cancer (HR = 0.35, 95% CI 0.17 to 0.73 and 0.71, 95% CI 0.56 to 0.91, respectively) [24].

A Danish registry-based case–control study of 13,241 ovarian cancers revealed a 42% reduction in ovarian cancer risk associated with bilateral salpingectomy (odds ratio (OR): 0.58; 95% CI 0.36 to 0.95) [25]. These findings are consistent with those of numerous epidemiological studies, which indicate that tubal sterilization is associated with a reduced risk of developing ovarian cancer. Furthermore, bilateral salpingectomy has been demonstrated to result in an even greater reduction in risk. However, it is crucial to acknowledge that the concept of tubal removal for cancer prevention was not established in any of these studies. The salpingectomy procedure was performed for a range of reasons, or primarily for unspecified reasons, which may have resulted in the observed results being influenced by unidentified confounding factors.

In a retrospective cohort study, a Canadian research group presented the results of a study in which opportunistic salpingectomy was intentionally performed for the first time as a method of cancer prevention [26]. The incidence of high-grade serous ovarian cancer was then compared in two different patient groups: those who underwent opportunistic salpingectomy during hysterectomy or bilateral salpingectomy as a method of tubal sterilization (*n* = 25,889) and those who underwent hysterectomy without salpingectomy or tubal ligation without salpingectomy (*n* = 32,080). Potential confounders that could have influenced the risk of ovarian cancer, such as parity or contraceptive use, did not differ significantly between the two groups. After a median follow-up of 7.3 years, no cases of HGSTOCs were observed in the first cohort, while 15 HGSTOCs were identified in the other cohort. The age-adjusted expected number in the salpingectomy cohort was 5.27 (95% CI 1.87–19.29), and the number of non-serous ovarian cancers was lower (five observed cases versus eight). The findings from retrospective studies corroborate the assertion that salpingectomy exerts an influence, albeit to a diminished degree, upon the progression of non-serous ovarian carcinomas. The potential mechanisms underlying the pathogenesis of these neoplasms may involve additional factors, such as endometriosis. The observed numbers of breast and colorectal cancers in this study did not exhibit significant disparities between the two groups and were consistent with the projected age-adjusted numbers (22.1 expected, 23 observed; 9.35 expected, 8 observed). This study is the first to demonstrate the feasibility of opportunistic salpingectomy as a primary prevention strategy for ovarian cancer: no patients who underwent bilateral salpingectomy between 2008 and 2017 developed HGSTOC.

## Risk reduction of ovarian cancer through other measures

It is crucial to acknowledge that the cohort of non-BRCA patients is not a homogeneous entity. A number of studies have identified a range of risk factors that should be taken into account when assessing the risk in this group. These factors include obesity, low physical activity, immunosuppression (e.g. following transplantation or in patients with diabetes mellitus), and long-term hormone replacement therapy [27]. These factors have the potential to influence the risk of developing ovarian cancer and should be given careful consideration when deciding on prophylactic measures such as opportunistic salpingectomy. In addition to the aforementioned specific risk factors, it is also important to consider the general perioperative risks as part of the shared decision-making process. A personalized, patient-centered approach that weighs the potential benefits and risks for each patient is essential for informed decision-making. Furthermore, lifestyle modifications, such as addressing obesity, promoting physical activity, and incorporating nutritional counseling, should also be included in the discussion with patients to support their health and minimize potential risk factors.

## Other potential benefits of opportunistic salpingectomy

A clinically recognized constellation is defined as the occurrence of a hydrosalpinx subsequent to the diagnosis of an initially ambiguous ovarian cystic finding many years after a hysterectomy without salpingectomy. A retrospective study conducted at a single center demonstrated that performing opportunistic salpingectomy can reduce the risk of re-surgical intervention for benign adnexal pathology in comparison to individuals who did not undergo the procedure (12.56% vs. 4.16%; *p* = 0.04). [28, 29]. Table 1 summarizes the potential risks and benefits.

**Table 1:** Potential benefits and risks of opportunistic Salpingectomy

Potential benefits:	Risks:
Reduction in ovarian cancer risk [30]	Surgical morbidity/adverse events? [27–29, 31–33]
Reduction in adnexal surgery following hysterectomy [25, 26]	Early menopause? [34–36]

## Morbidity of surgical procedures

The perioperative and postoperative complications of hysterectomy with opportunistic salpingectomy have been compared with those of hysterectomy without salpingectomy in numerous studies, and the results are largely consistent. There are no significant differences in blood loss, frequency of blood transfusions, postoperative fever, infections, or length of hospital stay regardless of the surgical approach, i.e., abdominal, vaginal, or minimally invasive [31].

The validity of some studies has been questioned due to the relatively small number of patients included and the occurrence of adverse events [32]. According to the authors of meta-analyses on this topic, the results are sometimes of moderate quality due to heterogeneous outcome measures and, in particular, different reading times with widely varying follow-up periods [33, 37]. A retrospective registry study’s primary strength lies in its capacity to amass a substantial number of cases. For instance, a Canadian registry study examined 10,697 cases of bilateral salpingectomy alongside 195,238 cases of hysterectomy alone. The findings revealed no statistically significant disparities in postoperative complications, infections, fever, or transfusions, which occurred at low frequencies, single-digit percentages, or per thousand [38].

However, the question remains as to whether supplementary surgical procedures that extend the operating time are consistently accompanied by an elevated risk of intraoperative or perioperative complications. However, apart from a slight increase in operative time (0 to 16 min [38, 39]), salpingectomy does not appear to be associated with any major general or specific complications associated with hysterectomy.

A comparison of bilateral salpingectomy at cesarean section with tubal ligation reveals discrepancies in outcomes between the two groups. A retrospective study compared 39,7260 patients who underwent bilateral salpingectomy between October 2015 and December 2018 with 203,400 patients who underwent tubal ligation during the same period. After excluding cases with cesarean section and concomitant hysterectomy, the bilateral salpingectomy group had a higher incidence of bleeding (3.4% vs. 3.0%; OR 1.16; 95% CI 1.06–1.26). In addition, the rate of oophorectomy was higher in the bilateral salpingectomy group than in the tubal ligation group (0.3% vs. 0.1%; OR 1.75; 95% CI 1.22–2.50) [40].

A Canadian retrospective cohort study compared 8440 patients who underwent bilateral salpingectomy with 9744 patients who underwent tubal ligation during cesarean section. The study’s findings indicated that salpingectomy was associated with a reduced incidence of perioperative complications, with no significant difference in postoperative complications observed between the two groups [41]. Consequently, salpingectomy should be considered as a replacement for tubal ligation during cesarean section as a preventive strategy for cancer.

## Ovarian perfusion and endocrine reserve after hysterectomy and opportunistic salpingectomy

A meta-analysis of 14 studies involving 1,457 premenopausal women found a statistically significant association between hysterectomy and earlier age at menopause [42, 43]. In the absence of hormone replacement, this may adversely affect bone health and increase the risk of cardiovascular events in older age. It is postulated that this phenomenon is due to impaired ovarian blood flow and loss of paracrine and endocrine signals from the uterus.

In the absence of menstrual bleeding as a clinical parameter of menopause, the measurement of serologic surrogate markers of ovarian reserve such as anti-Müllerian hormone (AMH), antral follicle count, follicle stimulating hormone (FSH), and luteinizing hormone (LH) is often used after hysterectomy. A comparison of serum levels in the control group, which did not undergo hysterectomy, showed a significant difference, indicating a negative effect of hysterectomy on ovarian reserve.

The evidence regarding the effect of additional bilateral salpingectomy performed at the time of hysterectomy on ovarian function remains inconclusive. Several comparative studies have monitored serum levels of ovarian endocrine function in the postoperative period. In the mesosalpinx, the anastomosing vessels are observed to form an arcade-like arrangement. During salpingectomy, it is recommended to spare these vessels by cutting the tube directly at the tube wall. This approach is designed to prevent reduction of ovarian blood supply by sparing the mesosalpinx vessels. A prospective, randomized trial was conducted to evaluate the effects of tubal sterilization or bilateral salpingectomy in patients between the ages of 35 and 50 with regular menstrual bleeding who elected bilateral salpingectomy as part of myomectomy surgery. In the standard arm, which included 91 patients, the fallopian tubes were removed at the posterior wall with preservation of the mesosalpinx. In the experimental arm, which included 95 patients, the tubes were removed completely, including the mesosalpinx. The primary endpoint of the study was the change in AMH levels from the preoperative period to the postoperative period three months later. A meticulous examination revealed no substantial disparities in preoperative characteristics or AMH levels between the two study groups. Consequently, no statistically significant variations were identified in the postoperative decline of AMH levels (ranging from −0.09 ± 0.24 to −0.07 ± 0.22 ng/mL; *P* = 0.54). Additionally, no statistically significant differences were observed between the two groups in other parameters, including hormone levels, gonadotropin levels, and Doppler ultrasound parameters [34].

A retrospective cohort study revealed that bilateral salpingectomy during hysterectomy was associated with an elevated risk of developing menopausal symptoms within 1 year post-surgery compared with hysterectomy without salpingectomy [35]. Patients were interviewed by the surgeon using validated questionnaires as part of a Swedish quality registry for gynecologic surgery. These interviews were conducted preoperatively and at 8 weeks and one year postoperatively. Patients were specifically asked about menopausal symptoms such as hot flashes, sweats, and heart palpitations. Prior to surgical intervention, no notable discrepancy was observed in the self-reported menopausal symptoms between the two study groups. However, at the 1-year postoperative interval, a significant increase in the incidence of postoperative menopausal symptoms was observed in the bilateral salpingectomy group compared to the hysterectomy-alone group (RR, 1.29; 95% CI 1.04–1.60; adjusted RR, 1.33; 95% CI 1.04–1.69).

However, a potentially relevant discrepancy was observed between the two groups in terms of patient characteristics. The mean age of the 3473 patients who did not undergo salpingectomy was 45.1 years (interquartile range [IQR] 42–49 years), which was significantly higher than the mean age of the 1433 patients who underwent hysterectomy and salpingectomy (44.1 years [IQR 41–48 years], *P* < 0.001). This observation suggests that the elevated risk of menopausal symptoms observed in the salpingectomy group may not be exclusively attributable to the surgical procedure itself, but may also be influenced by age. Given these considerations, it is challenging to draw definitive conclusions.

To address this knowledge gap, a meta-analysis was conducted to evaluate the potential impairment of ovarian reserve in patients who underwent opportunistic salpingectomy during abdominal surgery. The patients in this group were then compared to a control group. The surgical procedures included in the analysis were typically hysterectomy, myomectomy, or cesarean section. The no salpingectomy group consisted of nine prospective randomized trials with 24 to 104 patients, three prospective cohort studies with 38 to 50 patients, and three retrospective cohort studies with 50 to 373 patients. Serum levels were measured and compared preoperatively and between three weeks and six months postoperatively [36].

The analysis revealed no statistically significant variation in the mean decline of serum AMH (mean difference (MD): −0.07 ng/ml, 95% CI −0.18; 0.05), E2 (MD: 3.97 pg/ml, 95% CI −0.9; 2.86), FSH (MD: 0.33 mIU/ml, 95% CI −0.15; 0.81), and LH (MD: 0.03 mIU/ml, 95% CI −0.47; 0.53). The authors of the study conclude that salpingectomy does not significantly reduce ovarian reserve, at least in the short term. However, the extent to which ovarian reserve may continue to change over time or whether menopause may occur earlier despite constant postoperative anti-Mullerian hormone (AMH) levels remains a subject of ongoing debate [35].

The data do not provide compelling evidence that an additional salpingectomy in combination with a hysterectomy results in a notable reduction in ovarian reserve. A prospective study that systematically documents the onset of menopause after bilateral salpingectomy compared to patients who did not undergo salpingectomy is not yet available. However, two studies are currently collecting this data prospectively [44, 45].

## Clinical practice and future concerns

A review of surveys of surgeons and assessments of surgical procedures in hospital registries in several countries, including the United States, Canada, and Australia, shows an increase in the rate of opportunistic salpingectomy over the past decade [30, 46–49]. In Germany, the proportion of opportunistic salpingectomies performed during hysterectomy increased from 2% in 2005 to 45% in 2020. Both adnexa were removed in 27% and 31% of patients, respectively, while the proportion of hysterectomies without salpingectomy decreased from 71 to 24%. In 2020, the number of salpingectomies performed in Germany was approximately four times higher than in 2015 [50]. This finding suggests that opportunistic salpingectomy has become a routine procedure in Germany and a de facto standard for primary prevention of HGSTOC.

The efficacy of surgical prevention through organ removal is enhanced with increased frequency of application. Inclusion of patients undergoing other elective abdominal procedures, such as cholecystectomy, in the pool of potential candidates for opportunistic salpingectomy could facilitate its increased prevalence. Results from a comprehensive Markov model simulation of 1.2 million women in Germany over a lifetime horizon suggest that incorporating opportunistic salpingectomy into abdominal surgeries could reduce the incidence of ovarian cancer cases by up to 15% while yielding significant cost savings, with an estimated ICER (ICER, incremental costs per incremental LY gained) of €-8685.50 per QALY gained [51]. Technical aspects, including patient positioning and port placement, warrant careful consideration, and potential complications should be duly documented. A survey of 20 participants revealed that 12 would accept salpingectomy during a scheduled laparoscopic cholecystectomy, 7 would require additional time to make a decision but were not opposed, and 1 would refuse [52]. However, general surgeons would require specialized training to avoid any potential compromise to the ovarian blood supply and must possess the requisite skills to engage in complex patient counseling regarding the associated risks and benefits. It is recommended that experienced gynecologists be involved in both the counseling and surgical aspects of the procedure to ensure optimal outcomes. Further considerations pertain to the question of whether the procedure can be justified as a distinct surgical intervention and how its costs can be covered.

From an oncologic perspective, one of the most pressing issues is the safety of delayed oophorectomy in high-risk patients, i.e., prophylactic salpingectomy with initial preservation of the ovaries followed by oophorectomy. To this end, patients will be enrolled in trials such as TUBA-WISP II, in which 1500 BRCA1 and 1500 BRCA2 mutation carriers will be randomized to receive either RRSO or prophylactic salpingectomy alone with delayed oophorectomy [53]. Data are expected in 2036, after completion of enrollment in 2026 and a minimum follow-up of 10 years.

It is noteworthy that the study by Hanley Pearce et al. provides compelling evidence in support of this approach [26]. The study compared patients who underwent opportunistic salpingectomy with those who underwent hysterectomy alone or tubal ligation without salpingectomy. The study, which included 25,889 patients who underwent opportunistic salpingectomy, found no cases of high-grade serous ovarian cancer (HGSTOC) in this cohort. This is in contrast to the 15 cases observed in the comparator cohort. This remarkable result emphasizes the efficacy of opportunistic salpingectomy in reducing the risk of ovarian cancer and suggests that it could be a valuable addition to current preventive measures. Although these findings are promising, further prospective research is needed to fully understand the potential impact on ovarian reserve and to optimize implementation in different surgical settings.

In conclusion, it is recommended that opportunistic salpingectomy be offered during routine gynecologic surgery to all women who have completed their family planning. Further prospective studies are required to ascertain the extent of the reduction in ovarian cancer risk and to exclude any effects on menopause.

## Supplementary Information

Below is the link to the electronic supplementary material.

Supplementary file1 (DOCX 14 KB)

## Data Availability

No datasets were generated or analysed during the current study.
