# Supplementary material for: Intergroup statement: opportunistic salpingectomy—molecular pathology, clinical outcomes and implications for practice (German Ovarian Cancer Commission, the North-Eastern German Society of Gynecologic Oncology (NOGGO), AGO Austria and AGO Swiss)
Source: Arch Gynecol Obstet. 2025 Apr 2;311(5):1451–9. doi: 10.1007/s00404-025-07974-z (PMC12033089; doi:10.1007/s00404-025-07974-z)
Supplement: Supplementary file 1 — Supplementary file1 (DOCX 14 KB) [file 404_2025_7974_MOESM1_ESM.docx]

# **Recommendations :** It is crucial to acknowledge the constraints of the data employed in this review, which are predominantly based on retrospective studies. These studies hold great promise but do not represent the highest level of evidence. It is imperative to weigh the potential benefits of cancer prevention against the currently unverified risks associated with the intervention. In light of the extant evidence,, the Gynecological Oncology Associations of Austria, Switzerland and Germany (*Arbeitsgemeinschaft für Gynäkologische Onkologie der OEGGG, Arbeitsgemeinschaft der SGGG für Gynäkologische Onkologie, Kommission Ovar der AGO*) issue the following recommendations:

**Salpingectomy Recommendation**: For patients no longer considering childbearing, an opportunistic salpingectomy should be recommended as part of the surgery.

**Screening and Counseling**: Patients undergoing elective surgery should be screened for a family history of breast and ovarian cancer and referred for genetic counseling if needed.

**Informed Decision-Making**: A participatory decision-making process is crucial. Patients should be fully informed of the potential benefits, risks, and side effects of prophylactic salpingectomy, including perioperative complications and the possibility of early menopause.

**Lifestyle Modifications**: The potential need for lifestyle changes, including weight loss, physical activity, and nutritional counseling, should be discussed to promote overall health and well-being.

**Evaluation for STICs**: A systematic fallopian tube evaluation is recommended, using the SEE-FIM protocol. However, the necessity of this in all cases is debated due to the effort involved and the low prevalence of STICs in the average-risk population. Patients diagnosed with precursors can be registered in a new STIC registry (https://stic-register.idg-rlp.de).

**Contraceptive Approach**: Bilateral salpingectomy should be considered as the primary surgical method of contraception, replacing traditional approaches like tubal ligation.

Table 2 Recommendations
